# Optimized Antimicrobial Peptide Jelleine-I Derivative Br-J-I Inhibits Fusobacterium Nucleatum to Suppress Colorectal Cancer Progression

**DOI:** 10.3390/ijms24021469

**Published:** 2023-01-11

**Authors:** Fengjing Jia, Qun Yu, Ruolei Wang, Ling Zhao, Fuwen Yuan, Haidong Guo, Yunhui Shen, Feng He

**Affiliations:** 1 Academy of Integrative Medicine, Shanghai University of Traditional Chinese Medicine, Shanghai 201203, China; 2 School of Pharmacy, Shanghai University of Traditional Chinese Medicine, Shanghai 201203, China

**Keywords:** colorectal cancer, fusobacterium nucleatum, antimicrobial peptides, adjunctive therapy, antimicrobial treatment

## Abstract

Colorectal cancer (CRC) is a major health burden worldwide due to its high morbidity, mortality, and complex etiology. *Fusobacterium nucleatum* (*Fn*), a Gram-negative anaerobe found in 30% of CRC patients, promotes CRC carcinogenesis, metastasis, and chemoresistance. Effective antimicrobial treatment is an unmet need for the rising CRC burden. Antimicrobial peptides (AMPs) represent a new class of antimicrobial drugs. In our previous study, we did the structure-activity study of Jelleine-I (J-I) and identified several halogenated J-I derivatives Cl-J-I, Br-J-I, and I-J-I. To determine whether those J-I derivatives can be a new therapy for bacterial-associated CRC, here we tested the antibacterial activities of these AMPs against *Fn* and their effects on CRC development. We found that Br-J-I showed the highest anti-*Fn* activity and Br-J-I may target membrane-associated FadA for *Fn* membrane disruption. More importantly, *Fn* promoted the growth of CRC cells-derived xenograft tumors. Br-J-I suppressed *Fn* load, colon inflammation, and *Fn*-induced CRC growth. Of note, Br-J-I induced better anti-CRC effects than common antibiotic metronidazole and Br-J-I sensitized the cancer-killing effect of chemotherapy drug 5-fluorouracil. These results suggest that Br-J-I could be considered as an adjunctive agent for CRC treatment and AMPs-based combination treatment is a new strategy for CRC in the future.

## 1. Introduction

Colorectal cancer (CRC) is the most common malignant tumor of the digestive tract and the second leading cause of cancer-related deaths worldwide [1]. Lifestyle factors, such as prevalence of obesity, hyperlipidemia, physical inactivity, unhealthy diet, alcohol consumption, cigarette smoking, and dysbiosis, contribute to the increasing incidence and mortality of CRC [2,3]. Even though recent advances in early detection screenings and treatment options have reduced CRC mortality in developed nations, its incidence is steadily rising in both developed and developing nations [2]. The International Agency for Research on Cancer (IARC) estimates that the global burden of colorectal cancer will increase by 56% between 2020 and 2040 [1]. Furthermore, a rising incidence of early-onset CRC is emerging and poses a bigger global public health threat [4]. Early diagnosis of CRC is not very common, and most patients are diagnosed at an advanced stage. Advanced CRC is a significant cause of cancer mortality and the prognosis of advanced CRC is poor.

The ideal treatment for CRC is to achieve complete removal of the tumor and metastases, which mostly requires surgical intervention. However, most patients diagnosed with advanced state of CRC are not suitable for surgery. Chemotherapy is the most widely utilized tumor treatment and it helps to improve the disease-free survival and overall survival of patients. However, most chemotherapeutic drugs are associated with adverse effects due to their non-specific toxicities toward various types of rapidly proliferating and dividing cells in the body, such as hematopoietic stem cells in the bone marrow [5]. Furthermore, CRC often relapses after therapy and patients with relapsed/refractory CRC consistently exhibit even worse survival [6]. Therefore, there remains an unmet need for safe and effective treatments for CRC patients, especially for patients in advanced stages.

The human gut houses a large and complex micro-ecosystem, in which bacteria dominate. There are about 100 trillion microbes in the adult gut system, outnumbering human cells by 10 to 1 [7]. These microbes and the intestinal mucosal immune system constitute a complex intestinal microecology and imbalance of this microecology may lead to intestinal diseases and tumors. Pathogens often contribute to inflammation, which precedes malignant transformation, and are considered as contributors to CRC. Studies from metagenomics and next-generation sequencing found that the intestinal microecology of CRC patients exhibits dysbiosis and the flora of tumor tissues differs from those of paired normal tissues [8]. Those studies revealed that the amount of some bacteria is closely related to colorectal carcinogenesis [9]. Fusobacterium nucleatum (*Fn*), an anaerobic gram-negative oral commensal bacterium, was found in approximately 30% of all patients with CRC both in the intestinal microbiota but also in tumor epithelium [10,11]. *Fn* is extremely active in the altered intestinal microecology and is closely related to CRC initiation and progression [12,13,14]. *Fn* promotes CRC formation by stimulating the production of pro-inflammatory factors, such as IL-17 and TNF [11,15]. Virulence factors in *Fn* also contribute to colorectal carcinogenesis [13]. In addition, *Fn* assists CRC cells in escaping immune attack by inhibiting the cytotoxic activities of natural killer (NK) cells [16] and recruiting tumor-infiltrating myeloid cells [17]. Currently, the bacterial associated-CRC lacks effective bacterial-related drugs for CRC prevention and treatment.

The main treatments for abnormal intestinal flora are using biogenic, probiotics, fecal microbiota transplantation (FMT), and antibiotics. In the clinical study, high doses of prebiotics in a short period showed adverse effect on glucose metabolism [18]. The effects of prebiotics on the human body remain uncertain, which may be attributed to the diet habits and physical quality of subjects, as well as the dose of prebiotics intake. Probiotics and FMT are risky and may pose safety concerns [19,20]. Probiotics are beneficial to the human body by regulating intestinal flora, but they are not suitable for patients with immune deficiency and intestinal barrier dysfunction due to the potential infection and bacteremia induced by probiotics [21]. FMT may spread infectious bacteria from donors or antibiotic resistance genes from symbiotic bacteria [20]. In addition, the adverse effects of antibiotics may cause additional damage and adverse reactions in frail CRC patients [22,23]. With the extensive use of antibiotics, the emergence of a large number of antibiotic-resistant bacteria will further increase the difficulty of treatment. There is a lack of safe and effective treatment methods for intestinal bacteria. There is a huge space for the development of new drugs targeting pathogenic intestinal bacteria.

Antimicrobial peptides (AMPs) form an important component of host innate immunity in all living organisms against invading pathogens [24]. AMPs are bioactive molecules that are induced when the host is exposed to infected microorganisms to inhibit the pathogenic microorganisms. In recent years, AMPs have attracted extensive attention due to their low toxicity, good antibacterial effect, and low probability of resistance development [25,26,27,28]. In addition, peptide drugs fall in between small molecules and protein/antibody drugs with highly targeted specificity and no immunogenicity. However, low antimicrobial activity, poor metabolic stability, and short half-life usually limit the clinical applications of AMPs. Jelleine-I (J-I), first isolated from the royal jelly of honeybees, is a typical amphipathic AMP and shows broad antimicrobial spectrum [29]. We previously did the design and activity study of J-I and found that the halogenated derivatives of J-I, including Br-J-I, Cl-J-I, and I-J-I, showed potent antibacterial activity, increased proteolytic stability, and negligible cytotoxicity [30]. J-I halogenated derivatives possess great potential to be developed as a new antibacterial agent. In this study, we studied the effects of these halogenated J-I analogs on pathogenic intestinal flora, especially the cancer-promoting *Fn*, and the *Fn*-induced CRC development. We tested the antibacterial activity of these halogenated J-I analogs against *Fn* and the underlying mechanism. We found that, among these halogenated J-I analogs, Br-J-I showed the best antibacterial activity against Fn, and more importantly, Br-J-I effectively inhibited *Fn*-induced inflammation and CRC development. Br-J-I could be combined with the conventional anti-CRC drug 5-FU to improve the effects of CRC treatment.

## 2. Results

### 2.1. Br-J-I Shows Potent Antimicrobial Activity against Fn

**Figure 1:**
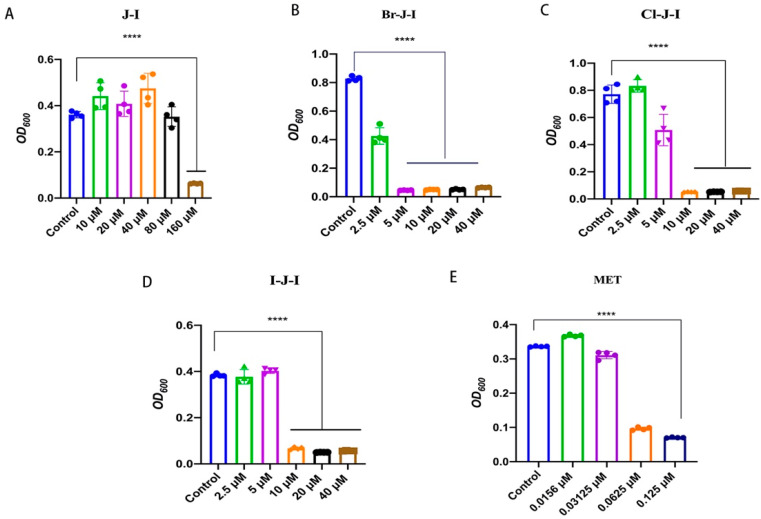
Antimicrobial activities of AMP Jelleine-I (J-I) and its derivatives against *Fusobacterium nucleatum* (*Fn*). Effects of Br-J-I (**A**), Cl-J-I (**B**), I-J-I (**C**), J-I (**D**), and Metronidazole (MET) (**E**) on the growth of *Fn* after 24 h treatment of different concentrations of AMPs or positive control MET. Each experiment was repeated at least three times independently. All values are represented as mean ± SEM. **** *p* < 0.0001, vs. Control.

We previously designed several new analogs of AMP J-I, aiming to search for a new J-I modifier with enhanced antimicrobial activity against some common pathogenic bacteria and we found that the halogenated derivatives of J-I, including Br-J-I, Cl-J-I, and I-J-I (Figure S1 and Table 1), showed potent antibacterial activity, increased proteolytic stability, and negligible cytotoxicity [30]. To further determine the potential clinical applications of these optimized J-I derivatives, we tested their activities on *Fn*, a common anaerobic gram-negative bacterium found in patients with intestinal microbiota dysbiosis, and their effects on the *Fn*-associated CRC. The MIC values and MBC values represent the antimicrobial activity of agents against bacteria in vitro. We first tested the antimicrobial activity against *Fn* of J-I and its halogenated derivatives, in which N-terminal Phe was replaced with chlorinated, brominated, and iodinated phenylalanine, respectively (Figure S1 and Table S1). Compared to J-I with an MIC of 160 μM, the MICs of Br-J-I, Cl-J-I, and I-J-I were 5 μM, 10 μM, and 10 μM, at least 10-fold increase in the anti-*Fn* potency (Table 2 and Figure 1). Among the halogenated derivatives, Br-J-I had the lowest MBC of 10 μM, compared with the 40 μM and 20 μM for Cl-J-I and I-J-I, respectively (Table 2). MBC of J-I was higher than 320 μM. The results showed that the halogenated J-I derivatives had better antimicrobial activity than J-I itself and Br-J-I had the best antimicrobial activity.

### 2.2. Br-J-I Induces Membrane Disruption of Fn to Inhibit Fn

**Figure 2:**
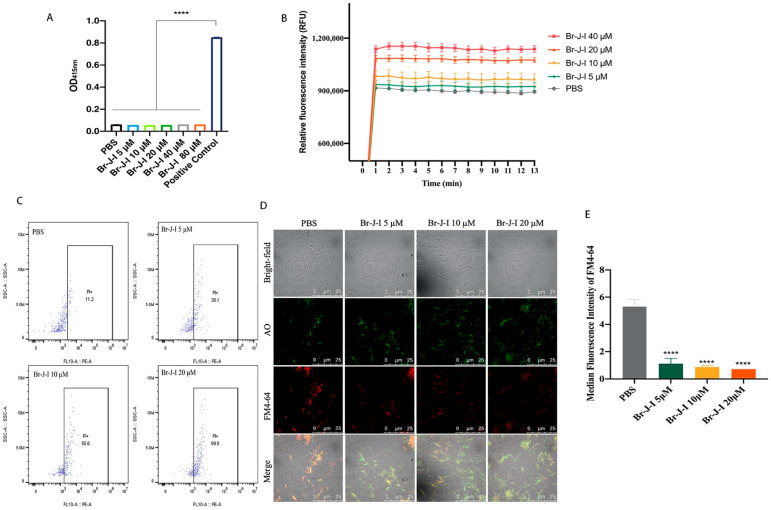
The mechanism of antibacterial activity of Br-J-I. (**A**) The effect of Br-J-I on the levels of intracellular hydrogen peroxide within *Fn.* (**B**) Dose dependent outer membrane permeabilization of *Fn* by Br-J-I*.* Time course of fluorescence of NPN bound to inner phospholipid membrane of *Fn* induced by different concentrations of Br-J-I. (**C**) Flow cytometry analysis of PI uptake of *Fn* induced by different concentrations of Br-J-I. (**D**) Confocal microscopic image of *Fn* co-stained with AO and FM4-64 with different concentrations of Br-J-I, scale bar: 25 μm. PBS was used as a negative control. (**E**) Quantitation of FM4-64 fluorescence intensity in C. PBS was used as a negative control. Each experiment was replicated at least three times independently. All values are represented as mean ± SEM. **** *p* < 0.0001, vs. *Fn* group.

**Figure 3:**
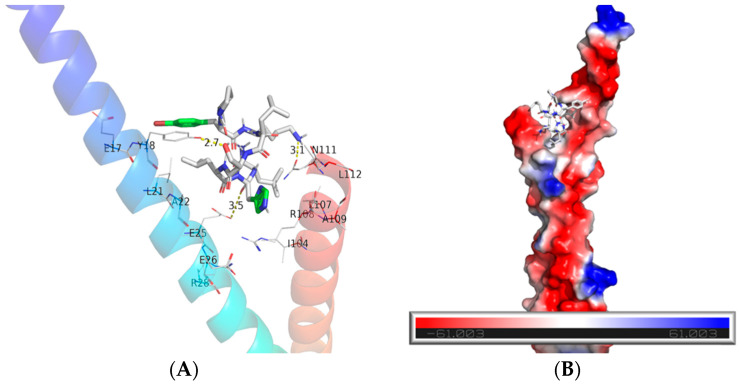
Molecular modeling of Br-J-I binding to potential target FadA. (**A**) Br-J-I interacts with the tail region of antiparallel α-helices of FadA (PDB: 3ETW) with the three potential hydrogen bonds shown in yellow dashed lines (distance labeled in Å). For clarity, only the protein segments that contain the groups of interest are shown. FadA is shown in an α-carbon rainbow cartoon from blue in N-terminus to Red in C-terminus. The potential interfacial residues for Br-J-I in FadA are shown in lines and Br-J-I are in sticks. N, O, and C atoms of side chains are colored blue, red and gray, respectively. Aromatic ring is in green and Br is in orange. (**B**) Surface representation of FadA illustrating the Br-J-I binding pocket. Hydrophobic, positively charged, and negatively charged surfaces are colored gray, blue, and red, respectively. Molecular docking was carried out by Vina docking software, and the interactions between ligand and receptor were analyzed and presented by PyMOL.

To understand how Br-J-I exerts bactericidal effect, we first tested whether Br-J-I induces H_2_O_2_ formation, which is a common mechanism contributing to the death of bacteria. Compared with positive control that kills bacteria by H_2_O_2_, Br-J-I did not alter intracellular level of H_2_O_2_ in *Fn* (Figure 2A). Then, we measured membrane integrity of *Fn* after treatment with Br-J-I, which is a common bacterial disruption mechanism for AMPs [31,32,33]. NPN is a hydrophobic fluorescent probe and its fluorescence intensity is weak in a hydrophilic environment, unless in a hydrophobic phospholipid membrane environment. NPN does not normally go to the inside of cells, because it cannot penetrate the cell outer membrane. When the outer membrane is damaged, NPN can access the cellular phospholipid membrane and fluoresce strongly. In contrast to background control fluorescence, strong NPN fluorescence was detected 1 min after Br-J-I addition and reached equilibrium (Figure 2B). Br-J-I induced increase of NPN fluorescence in a dose-dependent manner. To further detect the effect of Br-J-I on the membrane integrity, propidium iodide (PI) was used to stain the DNA of *Fn* in the presence of increasing concentrations Br-J-I and quantitated by flow cytometry. PI is membrane impermeable unless cell membranes are damaged. When PI binds to DNA, it produces strong red fluorescence. Br-J-I treatment significantly increased red fluorescent PI of *Fn* (Figure 2C). In addition, *Fn* was co-stained with lipophilic membrane-selective fluorescent dye FM4–64 and membrane permeable nucleic acid dye AO in the absence or presence of Br-J-I. In the absence of Br-J-I, FM4–64 was co-stained with AO (Figure 2D,E). When Br-J-I was present, fluorescence of FM4–64-stained *Fn* membrane was decreased (Figure 2D,E), thereby loss of co-staining of FM4–64 with AO, indicating that Br-J-I induced disruption of membrane integrity. The result suggested Br-J-I kills *Fn* by disrupting its membrane integrity. To further understand the mechanism of Br-J-I induced membrane disruption, we subsequently did molecular docking analysis between Br-J-I and structurally available bacterial membrane proteins with potential oligomerization domains. We found that *Fusobacterium* adhesin A (FadA), as the unique protein of *Fusobacterium*, is a potential binding target of Br-J-I. Br-J-I binds to the oligomerization domain of FadA with the binding free energy of −3.8 kcal/mol, and interfacial residues between them form three hydrogen bonds: one between S5 of Br-J-I and Y18 of FadA, the second one between K2 of Br-J-I and N111 of FadA, and the third one between H7 carboxyamide group of Br-J-I and E25 of FadA (Figure 3A). Br-J-I fits in an amphipathic groove of FadA with hydrophobic surface in the center and negative charged surface on the edge of groove (Figure 3B). FomA is a major outer-membrane protein of *Fn* [34] and AlphaFold prediction showed that FomA is structurally similar to multi-stranded anti-parallel beta-barrel containing a pore that aids the diffusion of small hydrophilic molecules across the outer membrane of Gram-negative bacteria [35,36]. Molecular docking revealed that Br-J-I interacts with FomA with the binding free energy of −6.6 kcal/mol (Figure S2). FomA has heat-modifiable oligomeric and conformational properties [34] and binding of Br-J-I to FomA may contribute to FomA stability and membrane permeability for *Fn* demise. FadA locates in the outer membrane of *Fn* and the molecular docking results suggest that Br-J-I likely targets FadA oligomerization motif and induces FadA oligomerization within membrane to permeabilize the membrane.

### 2.3. Br-J-I Exhibits Little Cytotoxicity to Colon Epithelial Cells and CRC Cells

**Figure 4:**
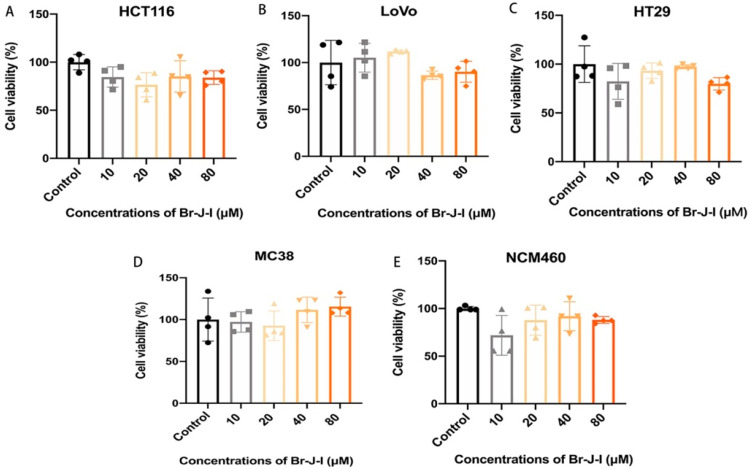
Cell viability of various CRC cell lines and colon epithelial cells in the presence of Br-J-I. Cell viability of human colon cancer cells HCT116 (**A**), LoVo (**B**), HT29 (**C**), murine colon cancer cells MC38 (**D**), and human colon epithelial cells NCM460 (**E**) treated with different concentrations of Br-J-I for 72 h.

Since Br-J-I displays potent anti-*Fn* activity, its cytotoxicity toward mammalian cells is one of the important factors that cannot be ignored. We used MTT assay to determine the cytotoxicity of Br-J-I to colon epithelial cells and colon cancer cells. As shown in Figure 4A–E and Figure S3, after 72 h incubation, Br-J-I, with the concentration up to 16 × MIC, did not show any significant observable effect on the viability of colon cancer cells HCT116, Lovo, HT29, MC38, and human colon epithelial cells NCM460. The results showed that Br-J-I had negligible cytotoxicity to human colon epithelial cells and colon cancer cells. This presents an advantage: that we can apply a dosage that specifically kills *Fn*, but with no cytotoxicity to mammalian cells.

### 2.4. Br-J-I Suppresses the Tumor-Promoting Effect of Fn

**Figure 5:**
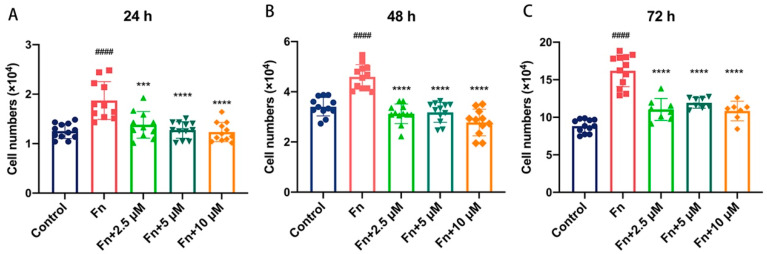
Effects of Br-J-I on the proliferation of human colon cancer cells co-cultured with *Fn.* Cell number analysis of human colon cancer cells HCT116 co-cultured with or without *Fn* in the presence of different concentrations of Br-J-I (0, 2.5, 5, and 10 μM) at 24 h (**A**), 48 h (**B**), and 72 h (**C**) to determine cell proliferation. Each experiment was independently performed three times. All data were shown as mean ± SEM. *** *p* < 0.001, **** *p* < 0.0001, vs. *Fn* group. #### *p* < 0.0001, vs. Control.

The effect of Br-J-I on cell proliferation of human colon cancer cells with *Fn* was determined by cell counting. HCT116 cells were co-cultured with *Fn* in the absence or presence of Br-J-I and the cell numbers were counted at 24 h, 48 h, and 72 h of incubation. *Fn* alone increased the cell proliferation of HCT116 cells and the cell numbers were dramatically increased from 24 h to 72 h (Figure 5A–C). When Br-J-I was present at 2.5 μM, 5 μM, and 10 μM, the *Fn* increased cell proliferation of HCT116 cells was significantly suppressed, to a level similar to that of the control group without *Fn* (Figure 5A–C). Of note, Br-J-I alone did not affect cell proliferation even at higher concentrations, as shown in Figure 4A. These results indicate that *Fn* enhanced the HCT116 proliferation and this effect was suppressed by Br-J-I.

### 2.5. Br-J-I Inhibits the Growth of CRC Induced by Intratumoral Fn in Mice Engrafted with HCT116

**Figure 6:**
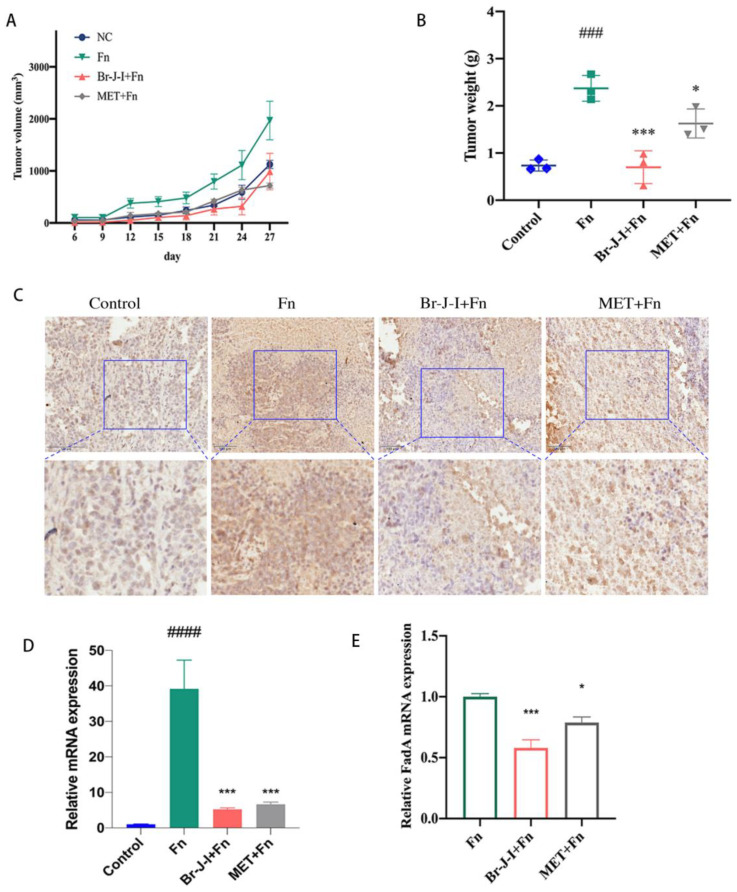
Effect of Br-J-I on the growth of the HCT116-xenograft tumors with intratumoral *Fn* colonization. Tumor volume (**A**) and tumor weight (**B**) of HCT116-derived tumors from the CRC mice inoculated with *Fn* in the presence of Br-J-I (10 mg/kg) or MET (40 mg/kg). (**C**) Immunohistochemistry of Ki-67^+^ cancer cells in the HCT116-tumor xenografts after the treatments of Br-J-I (10 mg/kg) and MET (40 mg/kg), respectively. Scale bar: 100 μm (Top), 4-fold magnification of top (Bottom). (**D**) Quantification of *Fn*-specific RNA in the tumors of CRC mice with or without *Fn* colonization in the presence of Br-J-I (10 mg/kg) and MET (40 mg/kg), respectively. (**E**) QPCR of FadA RNA in tumors of mice engrafted with HCT116 cells with *Fn* colonization and Br-J-I treatment. All values are represented as mean ± SEM. * *p* < 0.05, *** *p* < 0.001, vs. *Fn* group. ### *p* < 0.001, #### *p* < 0.0001, vs. Control.

To detect the effects of Br-J-I on the CRC tumor growth induced by *Fn* in vivo, CRC cells HCT116 were engrafted into mice and when tumors were formed, *Fn* was intratumorally colonized and different doses of Br-J-I were intraperitoneally administrated into mice every three days. Compared with the control group, *Fn* increased the CRC growth rate, evidenced by 100% increases in tumor size and tumor weight (Figure 6A,B). Both Br-J-I (10 mg/kg) and positive control metronidazole (MET, 40 mg/kg) suppressed the tumor-promoting effects of *Fn* and the tumor-inhibitory role of Br-J-I was better than that of MET (Figure 6A,B). In agreement with this, tumor proliferation marker Ki-67 was stained more in the *Fn*-treated CRC xenograft tumors, less in the group of *Fn* plus Br-J-I or group of *Fn* plus MET (Figure 6C). These results indicated that Br-J-I effectively inhibits the CRC tumor-promoting effect of *Fn*.

*Fn* load of the tumor tissues of CRC mice with *Fn* colonization was quantitated to further analyze whether Br-J-I suppresses the CRC growth by directly blocking *Fn* in tumors. As shown in Figure 6D, *Fn*-specific RNA was not detected in tumor tissues of CRC mice without *Fn* inoculation. After *Fn* inoculation, *Fn*-specific RNA in tumor tissues of CRC mice was dramatically increased. When Br-J-I was administrated, *Fn*-specific RNA was significantly reduced in tumor tissues of CRC mice with *Fn* colonization, similar to metronidazole (MET). In addition, Br-J-I inhibited the expression of the key *Fn* gene FadA within CRC xenografts (Figure 6E). These results suggest that Br-J-I suppresses the increased growth of CRC tumors enhanced by *Fn*.

### 2.6. Br-J-I Inhibits Fn-Induced Inflammation

**Figure 7:**
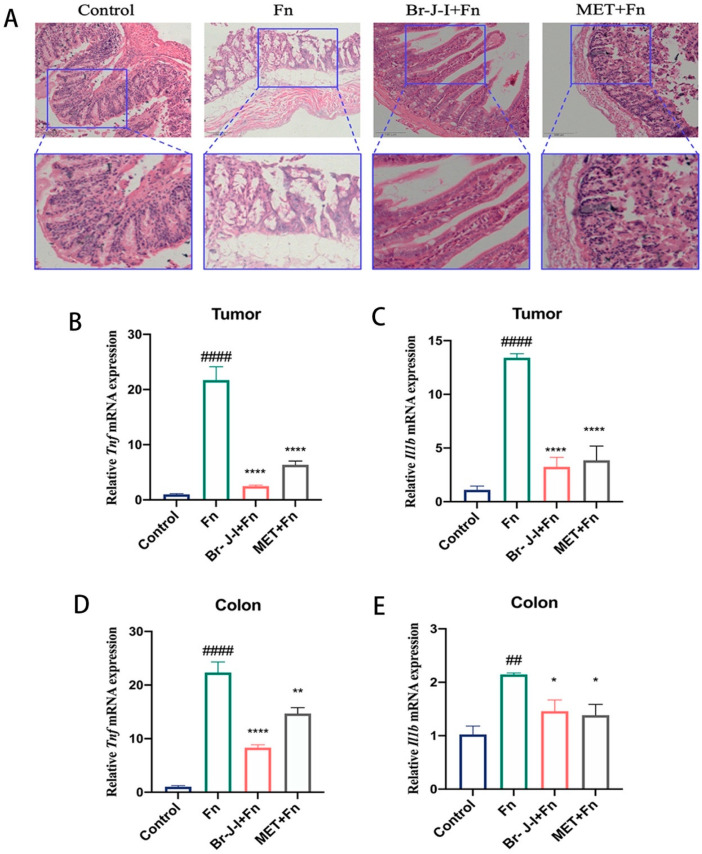
Effect of Br-J-I on the *Fn*-induced inflammation in vivo. (**A**) H&E staining of colon tissues of HCT116-xenograft-bearing mice to examine the gut inflammation and barrier. Scale bar: 100 μM (Top), 4-fold magnification of top (Bottom). (**B**–**E**) Quantification of the expression of genes encoding the proinflammatory cytokines TNF-α (**B**,**D**) and IL-1β (**C**,**E**) in the tumors (**B**,**C**) and the colon tissues (**D**,**E**) of the CRC cells-engrafted mice with or without *Fn* colonization in the presence of Br-J-I (10 mg/kg) and MET (40 mg/kg). All values are represented as mean ± SEM. * *p* < 0.05, ** *p* < 0.01, **** *p* < 0.0001, vs. *Fn* group. ## *p* < 0.01, #### *p* < 0.0001, vs. Control.

Bacteria-induced inflammation is closely related to CRC progression [37,38]. To further examine the inhibition of *Fn* by Br-J-I, H&E staining was used to determine the effect of Br-J-I on the *Fn*-induced inflammation in vivo. *Fn* administration altered colon histology and damaged the gut barrier (Figure 7A). When Br-J-I was administrated, the inflammation of colon tissues in CRC mice xenografts with *Fn* colonization was reduced compared to the *Fn* group (Figure 7A). To further verify the effect of Br-J-I on the inhibition of *Fn*-induced inflammation, the expression of key inflammatory cytokines TNF-α and IL-1β in the tumors and colon tissues of CRC mice xenografts was quantitated. As shown in Figure 7B–E, the expression of genes encoding the proinflammatory cytokines TNF-α and IL-1β in tumors and colon tissues were dramatically increased after *Fn* inoculation. Br-J-I decreased the expression of *Tnf* and *IL1b* mRNA in both tumors and colons of CRC mice with *Fn* colonization. The Br-J-I exhibited more *Tnf* inhibitory effect than MET, while Br-J-I and MET showed similar inhibitory effects on IL1b. These results indicated that Br-J-I inhibits *Fn*-associated CRC growth by directly suppressing *Fn* and *Fn*-induced inflammation.

### 2.7. Br-J-I Improves Intestinal Mucosa Tight Junction (TJ) in HCT116 Cell-Engrafted Mice with Intratumoral Fn Colonization

**Figure 8:**
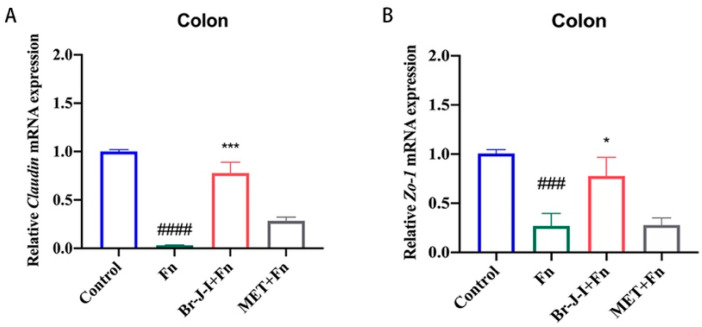
Effect of Br-J-I on the integrity of intestinal mucosa in murine engrafted with CRC and *Fn*. (**A**,**B**) Quantification of the effect of Br-J-I on the expression of genes encoding the tight junction proteins Claudin (**A**), and ZO-1 (**B**) in the colon tissues of the CRC cells-engrafted mice with or without *Fn* colonization in the presence of Br-J-I (10 mg/kg) and MET (40 mg/kg). Data are expressed as mean ± SEM. * *p* < 0.05, *** *p* < 0.001, vs. *Fn* group. ### *p* < 0.001, #### *p* < 0.0001, vs. Control.

Intestinal mucosa plays an important role in the absorption of nutrients and drugs. The intestinal mucosal barrier is closely related to the TJ, including the expression of key TJ proteins Claudin and ZO-1 [39]. To further test whether Br-J-I affects intestinal mucosal barrier, the effect of Br-J-I on the expression of TJ proteins Claudin and ZO-1 was detected. After inoculation with *Fn*, the expression of mRNAs encoding TJ proteins Claudin and ZO-1 were reduced significantly (Figure 8A,B). Br-J-I administration rescued the expression of Claudin and Zo-1mRNAs in intestinal tissue of CRC mice within *Fn* colonization, to a level similar to the control without *Fn*, indicating the improvement of intestinal barrier by Br-J-I. The results suggest that Br-J-I enhances the intestinal mucosal barrier by up-regulating the expression of key TJ proteins. Compared with MET, Br-J-I exhibited better anti-*Fn* effects in vivo and improved the intestinal mucosal barrier.

### 2.8. Br-J-I Synergizes with 5-FU to Exert the Antitumor Effect

**Figure 9:**
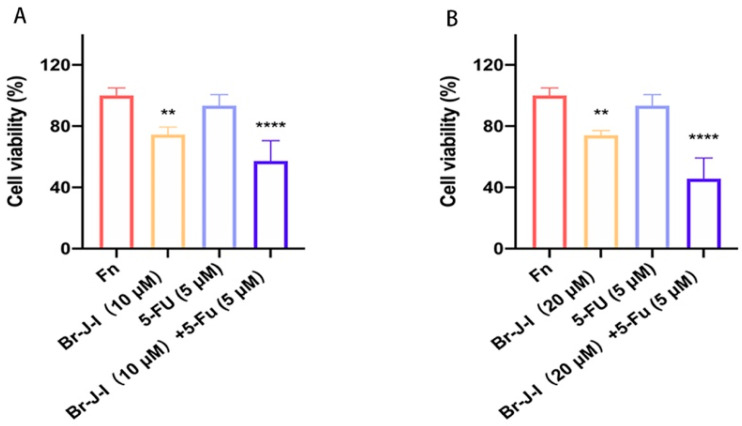
Br-J-I synergizes with 5-FU to inhibit proliferation of HCT116 cells. The HCT116 cells co-cultured with *Fn* were treated with *5*-FU (5 μM) alone or in combination with different concentrations of B-J-I for 48 h and MTT assay was conducted to determine cell viability. (**A**) 10 μM B Br-J-I /5 μM 5-FU, (**B**) 20 μM B Br-J-I /5 μM 5-FU. Results were representative of three independent experiments performed in four replicates and were expressed as mean ± SEM. ** *p* < 0.01, **** *p* < 0.0001, vs. *Fn* group.

Br-J-I does not act directly on the tumor cells, it inhibits the CRC progression induced by *Fn* and *Fn*-associated cancer-promoting inflammation. The 5-FU-based chemotherapy is a common treatment for CRC. However, clinical outcomes of 5-FU for CRC patients need to be improved, due to the emergence of relapse and refractory in CRC patients [40]. The effect of Br-J-I combined with 5-FU chemotherapy on the antitumor activity against CRC cells co-cultured with *Fn* was detected. At the concentration of 5 μM, 5-FU exhibited little cytotoxicity against HCT116 cells (Figure 9A,B). In line with Figure 5A–C, Br-J-I alone inhibited the tumor proliferation induced by *Fn*, shown as about 20% decrease in viability by MTT assay at 10 μM. When combined with Br-J-I, the cell proliferation of HCT116 cells was further reduced by about 40%. The Q values of the combination of Br-J-I (20 μM) with 5-FU (5 μM) were over 1.15, indicating a strong anti-tumor synergistic effect. The results suggested that the combination of Br-J-I and 5-FU exerts a better antitumor effect.

## 3. Discussion

At present, the clinical treatment of CRC mainly focuses on the tumors or the host itself, and the efficacy of the treatments is far from satisfactory. The existing treatment option cannot meet the needs of various CRC patients. *Fn* has been found to be abnormally increased in tumor tissues of CRC patients, which is one of the driving forces for the initiation and development of CRC [10,41,42]. However, safe and efficient treatment options for intestinal bacteria are very scarce, and the development of novel agents targeting intestinal bacteria is in urgent need.

AMPs are a kind of bioactive peptide produced in organisms, widely existing in insects, plants, and animals. As an important part of the innate immune defense, AMPs have the potential to be developed as new antimicrobials [43]. AMPs have a broad spectrum of antimicrobial activity and they usually kill microbes by disrupting cell membranes, through which they are less likely to develop resistance. AMPs often have low cytotoxicity to mammalian cells. In addition, AMPs have potent antitumor effects [44]. Due to these advantages, including potent antimicrobial activity, low toxicity, and difficulty in developing resistance, AMPs have become one of the hot spots in the research and development of novel antimicrobial agents.

In this study, AMPs were used to study the effects on the growth of CRC by regulating intestinal bacteria. The clinical studies have shown that the intestinal flora abnormally increased in CRC patients are mainly Gram-negative bacteria [11,12,14]. Our previous studies showed that AMP J-I derivatives, Br-J-I, Cl-J-I, and I-J-I showed potent antibacterial activity against Gram-negative bacteria [30]. In addition, these peptide analogs have very low cytotoxicity and good selectivity. In this study, the antibacterial activities of these peptide analogs against *Fn* were detected. The results showed that Br-J-I had the best antibacterial activity among these J-I derivatives. Subsequently, the antimicrobial mechanism of Br-J-I was studied. By studying membrane integrity, we found that the fluorescence of membrane-selective dye FM4–64 was decreased, indicating that Br-J-I disrupted the integrity of the bacterial cell membrane. Therefore, after Br-J-I treatment, extracellular fluorescent probe, such as PI, can permeate *Fn* and bind with DNA to produce strong fluorescence. The results showed that Br-J-I killed *Fn* by inducing the cell membrane disruption, which was consistent with the images of scanning electron microscopy of Br-J-I and other J-I derivatives against *Escherichia coli* (*E. coli*) in our previous study [30]. Electron microscopy revealed that addition of Br-J-I induced the formation of protrusions in the bacterial membrane and, eventually, disassembled the bacterial membrane [30]. In in vitro assay, we did not see the oligomerization property of Br-J-I and Br-J-I is not likely a transmembrane helix due to its short length and its amino acids distribution. Br-J-I could target bacterial membrane proteins and upon the binding of Br-J-I, the confirmational changes and oligomerization of complex could mediate the formation of bacterial membrane protrusions. FadA locates in the outer membrane of bacteria, which is a potential binding target of Br-J-I by molecular docking analysis. Br-J-I might target FadA oligomerization motif and induce FadA oligomerization within the membrane to permeabilize the membrane. Moreover, recent studies have proved that Gram-negative bacteria such as *Fn* and *E. coli* are closely associated with the initiation and development of CRC [14]. Our previous studies showed that Br-J-I exhibited potent antibacterial activity against *E. coli* [30]. High cytotoxicity is one of the factors limiting the widespread application of antibacterial agents. Although some antibiotics have excellent antibacterial activity, their high toxicity to host cells is unsuitable for frail and vulnerable CRC patients. In addition, the cytotoxicity of Br-J-I on human colon epithelial cells, human colorectal cancer cells, and murine colon cancer cell lines was minimal, and no significant cytotoxicity was found even at high doses (>8 MIC). These results suggest that Br-J-I will be tailor-made for CRC.

*Fn* promoted the proliferation of CRC cells, while the treatment of Br-J-I significantly rescued the proliferation of CRC cells by inhibiting *Fn*. The in vivo studies verified that *Fn* promoted the growth of CRC tumors and when Br-J-I was administrated, it significantly hindered the growth of CRC tumors by inhibiting Fn. We further quantified *Fn* load in the tumor tissue and showed that Br-J-I significantly reduced *Fn* load in the xenograft tumors in mice within *Fn* colonization. Consistently, the expression of FadA in tumors and colons was also reduced by Br-J-I. These results indicated that Br-J-I inhibited the *Fn*-induced growth of CRC tumors by directly blocking *Fn*. Moreover, our results showed that Br-J-I blocked the production of proinflammatory cytokines induced by *Fn*. This has important implications for the treatment of CRC, because CRC growth can be inhibited by suppressing the proinflammatory cytokines [38,45,46].

Br-J-I has potent antimicrobial activity and no cytotoxicity to CRC itself, and it significantly inhibits the growth of CRC promoted by *Fn*, suggesting that Br-J-I counteracts the adverse effects of *Fn* on CRC through its antimicrobial activity against Fn. The results indicated that the effect of Br-J-I on CRC tumors was mainly due to an indirect effect. 5-FU-based chemotherapy is a common treatment for CRC. Resistance to 5-FU often also emerges and clinical outcomes of 5-FU for CRC treatment is urgently needed for improvement [47]. At the concentration of 5 μM, 5-FU exhibited little cytotoxicity against HCT116 cells. After combination with Br-J-I, the cell viability of HCT116 cells was dramatically reduced by more than 40%. This suggests that Br-J-I sensitizes the cytotoxicity of 5-FU and Br-J-I synergizes with 5-FU to exert a better antitumor effect. Therefore, Br-J-I could be an ideal adjunctive therapy for CRC.

## 4. Materials and Methods

### 4.1. Synthesis and Purification of AMPs

AMPs were synthesized by a stepwise solid-phase method on rink amide 4-methyl-benzhydryl amine (MBHA) resin by N-9-fluorenylmethoxycarbonyl (Fmoc) chemistry, as described [48]. AMPs were purified by reverse phase high-performance liquid chromatography (RP-HPLC) (Waters 600, Milford, MA, USA) and gradient elution by 20–80% CH_3_CN/H_2_O with μBondapak C18 19 mm by 300 mm column [30]. The purity of these peptides is more than 95% determined by NMR.

### 4.2. Minimum Inhibitory Concentration (MIC) Assay

MICs of J-I and its halogenated derivatives against *Fusobacterium nucleatum* (*Fn*) (ATCC 25586) were determined by the Clinical and Laboratory Standards Institute microdilution method [30]. *Fn* was cultured in brain heart infusion broth (BD Difco, Cockeysville, MD, USA) at 37 °C under the anaerobic workstation (Don Whitley Scientific, Bingley, UK). Bacteria were cultured to logarithmic growth phase and adjusted to the inoculum size of 3 × 108 colony-forming units (CFU)/mL. Bacterial inoculum was incubated with various concentrations of AMPs or positive control metronidazole (MET) at 37 °C for 24 h. Each experiment was independently replicated at least three times.

### 4.3. Minimum Bactericidal Concentration (MBC) Assay

MBC of J-I and its halogenated derivatives were determined based on their MIC. The mixture with peptide analogs at different concentrations above the MIC was added to agar plates and then incubated at 37 °C. After 24 h, the number of CFU was observed [30]. The concentration that decreased the viability of *Fn* by ≥99.9% was defined as MBC of J-I and its halogenated derivatives.

### 4.4. Outer Membrane (OM) Permeability

The effect of Br-J-I on the OM permeability of *Fn* was determined by hydrophobic fluorescent probe N-Phenyl-1-naphthylamine (NPN, Aladdin, Shanghai, China) as previously described [30]. *Fn* was washed and suspended with PBS. The bacterial inoculum was adjusted to the OD 600 nm (OD_600_) of 0.5 ± 0.02 and divided into two groups: (1) peptides groups (100 μL *Fn* solution + 50 μL peptide+ 50 μL NPN); (2) Control group (100 μL *Fn* solution + 50 μL PBS + 50 μL NPN). The concentration of peptides is 1 × MIC to 8 × MIC. NPN fluorescence was continuously monitored for 13 min using a multifunctional microplate reader (Molecular Devices, Shanghai, China) with the excitation and emission wavelength of 350 nm and 420 nm, respectively.

### 4.5. Flow Cytometric Analysis

*Fn* was washed and suspended in PBS. The OD_600_ of bacterial inoculum was adjusted to 0.4 and treated with Br-J-I with the concentrations from 1 × MIC to 4 × MIC at 37 °C. After 5 min, the mixture was washed and suspended in PBS, followed by addition of 1 μL propidium iodide (PI, 1 mg/mL) for 15 min [49]. The effect of AMPs on the membrane permeability of *Fn* was conducted by using flow cytometer (Becton Dickinson and Company, Franklin Lakes, NJ, USA).

### 4.6. Laser Scanning Confocal Microscopy (LSCM)

*Fn* was washed and suspended in PBS with an OD_600_ of 0.4. The bacterial solution was treated with Br-J-I at the concentrations from 1 × MIC to 4 × MIC at 37 °C. After 1 h, the mixture was washed and resuspended in PBS containing 1 μL membrane-selective red fluorescent dye FM4-64 (1 μg/mL) and cell-permeant nucleic acid binding dye acridine orange (AO, 1 μg/mL) to stain the nucleic acids for 15 min. The fluorescence imaging was conducted by LSCM (Leica TCS SP8, Nussloch, Germany).

### 4.7. Binding Mode Prediction

The sequence and structure of the peptide was drawn by ChemDraw software to obtain the 2D structure of the peptide and the 2D structure of the peptide was then imported into Chem3D software to transform it into a 3D structure of the peptide according to the default parameters of the software [50]. The 3D structure of the peptide was used for the molecular docking analysis. The binding model of Br-J-I with *Fn* was predicted as formerly described [51]. The crystal structures of key bacterial proteins FadA (PDB: 3ETW) were downloaded from the Protein Data Bank. The structures of FomA were downloaded from AlphaFold prediction database with the code AF-Q47903 [36,52]. Molecular docking was performed by Vina docking software v.1.1.2. (http://vina.scripps.edu/). The interaction between ligand and receptor was determined by PyMOL software v1.7.2.1 (http://www.pymol.org/2/) to obtain information on binding energy and hydrogen bond.

### 4.8. Cytotoxicity

The cytotoxicity of Br-J-I was determined by 3-(4,5-Dimethylthiazol-2-yl)-2,5-diphenyltetrazolium bromide (MTT) assay (Beyotime, Jiangsu, China), as previously described [53]. Human CRC cell lines HCT116 (RRID: CVCL_0291), HT29 (RRID: CVCL_A8EZ), LoVo (RRID: CVCL_0399), and human colon epithelial cell line NCM460 (RRID: CVCL_0460) were purchased from the Shanghai Institutes for Biological Sciences of the Chinese Academy of Sciences. Murine colon cancer cell line MC38 was purchased from the Chinese Academy of Medical Sciences. The cells were cultured in Dulbecco’s modified Eagle medium (Gibco, Grand Island, NY, USA) supplemented with 10% Fetal Bovine Serum (Gibco, Grand Island, NY, USA), penicillin, and streptomycin at 37 °C with 5% CO_2_. 1 × 10^4^ cells were seeded per well in a 96-well plate and cultured overnight before addition of various concentrations of Br-J-I. After 72 h incubation with the AMPs, 20 μL MTT solution (5 mg/mL) was added to each well and incubated at 37 °C for 4 h [54]. The supernatant was discarded, and 150 μL dimethyl sulphoxide was added. The absorbance at 570 nm was detected by a microplate reader (Molecular Devices, Shanghai, China). The experiments were repeated 3 times. All human cell lines have been authenticated using STR (or SNP) profiling within the last three years. All experiments were performed with mycoplasma-free cells. All cell lines used was provided from American Type Culture Coll.

### 4.9. Effect of Br-J-I on Cell Proliferation

The Effect of Br-J-I on cell proliferation of colorectal cancer co-cultured with *Fn* was determined as previously described [55,56]. Human colorectal cancer cell lines HCT116 were seeded in a 24-well plate at a density of 1 × 104 cells per well. Cells were incubated with *Fn* (multiplicity of infection of 1000) in the presence and absence of various concentrations of Br-J-I. Cell counts for each group were conducted at 24 h, 48 h, and 72 h by Countstar cell counter (ALIT, Shanghai, China). Each experiment was independently replicated at least three times.

### 4.10. Effect of Br-J-I on the Growth of Murine CRC with Fn Colonization

The murine CRC with bacterial colonization was conducted as previously described [41,57]. Mouse experiments were performed under the National Guidelines for Animal Usage in Research of China and the guideline of the Ethics Committee of Shanghai University of Traditional Chinese Medicine with the approved protocol PZSHUTCM220711010. All NU/NU nude mice were purchased from the Shanghai Slac laboratory animals center. Human colorectal cancer cell HCT116 was subcutaneously injected into the right axilla of 4-week-old nude mice (1 × 10^7^ cells /100 μL PBS of each mouse) to establish the CRC xenograft model. Mice were randomly divided into 4 groups: (a) Saline as a control group; (b) *Fn* group; (c) Br-J-I (10 mg/kg) +*Fn*; (d) metronidazole (MET, 40 mg/kg positive group) +*Fn*. Nine days after HCT116 inoculation, *Fn* or vehicle control was intratumorally injected into each mouse every 3 days for 18 days. In addition, on the same day, mice were intraperitoneally administrated with Br-J-I or MET for the selected group every 3 days for 18 days. Tumor volume = (A × B^2^) /2, where A and B are the length and width and measured every 3 days. After the last administration, the mice were sacrificed for the collection of tumors and colon tissues. Tumors and colon tissues were fixed in 10% formalin, and then embedded in paraffin. Tumor sections were stained with Ki-67 monoclonal antibody (1:200, Servicebio, Wuhan, China) and colon tissues were stained with hematoxylin–eosin (H&E) (Solarbio, Beijing, China).

### 4.11. Quantification of Fn Load in the Tumors and the Expression of the Proinflammatory Cytokines in Tumors and Colon Tissues

Quantitative real-time polymerase chain reaction (qPCR) was used to quantitate *Fn* load in the tumors [10,17] and the expression of the proinflammatory cytokines in tumors and colon tissues as previously described [41,58,59]. Total RNAs were extracted from CRC tumors or colons using Trizol reagent (Beyotime, Shanghai, China) according to the manufacturer’s protocol. Hifair^®®^IIISuperMix Plus (Yeasen, Shanghai, China) and Hieff^®®^ qPCR SYBR Green Master Mix (Yeasen, Shanghai, China) were used to do the reverse transcription and qPCR, respectively, according to the manufacturer’s instructions. Relative mRNA expression of different groups was calculated by 2^(−ΔΔCT)^ method. The sequences of primers were listed in Table S1.

### 4.12. Analysis of Intestinal Permeability of Murine CRC

The effect of Br-J-I and MET on the expression of tight junction proteins Zona occludens protein 1 (ZO-1) and Claudin in the intestinal tissue of CRC-engrafted mice inoculated with *Fn* were analyzed by qPCR as described above.

### 4.13. Effect of Br-J-I Combined with *5*-Fluorouracil

Cell viability was measured by MTT assay. HCT116 cells (5000 cells/well) were seeded in a 96-well plate in the presence or absence of *Fn* (multiplicity of infection of 1000) and treated with different concentrations of Br-J-I and 5-fluorouracil (FU) (5 μM) for 48 h. The medium was carefully discarded, and RPMI1640 medium and 20 μL MTT solution (5 mg/mL) were added to incubate for 4 h. The medium was discarded and replaced with 150 μL dimethyl sulphoxide (DMSO). The absorbance was measured at 570 nm by a microplate reader (Molecular Devices, Shanghai, China). The synergistic effect of Br-J-I and 5-FU against HCT116 cells was estimated by Q value [60]. The Q value was calculated by the equation: Q = E (A + B)/E(A) + E(B) − E(A) × E(B) [61], where E(A + B) means the tumor growth inhibition rates of the combination of Br-J-I and 5-FU, E(A) and E(B) represent the tumor growth inhibition rates of Br-J-I and 5-FU, respectively. Q < 0.85 suggests an antagonistic effect of Br-J-I and 5-FU. 0.85 ≤ Q < 1.15 suggests an additive effect of Br-J-I and 5-FU. Q ≥ 1.15 suggests a synergistic effect of Br-J-I with 5-FU.

### 4.14. Statistical Analyses

Graphpad prism 8.0.2 software was used for data analysis and graphing. Statistical significance was evaluated by one-way analysis of variance (ANOVA with Dunnett’s post-hoc analysis). All values are expressed as mean ± SEM, and values of *p* < 0.05 were considered statistically significant. *p < 0.05, **p < 0.01, ***p < 0.001, ****p < 0.0001, compared with *Fn* group. #*p* < 0.05, ## *p* < 0.01, ### *p* < 0.001, #### *p* < 0.0001, compared with the NC group.

## 5. Conclusions

In summary, the optimized AMP Br-J-I, designed by us, exhibits potent antibacterial activity against *Fn* with negligible cytotoxicity. Br-J-I effectively inhibited Fn-induced tumor-promoting effect of CRC and inflammation by directly killing Fn, which has the potential to become a novel therapy for regulating intestinal bacterial dysbiosis and an adjunct therapy for CRC. Several intestinal bacteria, including Fn, are abnormally active in CRC patients, posing a great risk of colorectal carcinogenesis, cancer metastasis, and chemoresistance. However, safe and efficient treatment options for intestinal bacteria are very scarce in fragile CRC patients. This study provides a novel approach for the clinical treatment of CRC, in which AMPs can be used as adjuvant therapy to eliminate the adverse effects of pathogenic intestinal bacteria on CRC. In addition, Br-J-I synergizes with 5-FU to exert the antitumor effect, proving that Br-J-I as adjunctive therapy would further enhance the chemotherapy efficacy of CRC. We hope that this study will provide a new direction for the future development of candidates for adjunctive therapy for CRC.

## Figures and Tables

**Table 1:** Amino acid sequence of J-I, Br-J-I, Cl-J-I, and I-J-I.

Name	Peptides Sequence
J-I	PFKLSLHL-NH_2_
Br-J-I	PF^a^KLSLHL-NH_2_
Cl-J-I	PF^b^KLSLHL-NH_2_
I-J-I	PF^c^KLSLHL-NH_2_

a, 4-Br-phe-amino acid; b, 4-Cl-phe-amino acid; and c, 4-I-phe-amino acid.

**Table 2:** MIC and MBC of peptides against *Fn*.

Name	Br-J-I	Cl-J-I	I-J-I	J-I	MET
MIC	5 μM	10 μM	10 μM	160 μM	0.125 μM
MBC	10 μM	40 μM	20 μM	320 μM	0.5 μM

MET: Metronidazole.

## Data Availability

The data that support the findings of this study are available from the corresponding author upon reasonable request.

## Supplementary Materials

The supporting information can be downloaded at: https://www.mdpi.com/article/10.3390/ijms24021469/s1.

## Abbreviations

AO: Acridine Orange; Antimicrobial peptides (AMPs); CFU: colony forming units; CRC: Colorectal cancer; CT: cycle threshold; DMSO: dimethyl sulfoxide; FadA: Fusobacterium adhesin A; 5-FU: 5-Fluorouracil; *Fn*: *Fusobacterium nucleatum*; H&E: hematoxylin–eosin; IL-1β: interleukin-1β; Jelleine-I (J-I); MBC: Minimum bactericidal concentration; MET: Metronidazole; MIC: Minimum inhibition concentration; MOI: multiplicity of infection; MSI: microsatellite instability; NPN: N-Phenyl-1-naphthylamine; PI: Propidium Iodide; qPCR: quantitative polymerase chain reaction; TNF-α: tumor necrosis factor α; ZO-1: Zonula occludens protein 1.
