# Supplementary material for: Optimized Antimicrobial Peptide Jelleine-I Derivative Br-J-I Inhibits Fusobacterium Nucleatum to Suppress Colorectal Cancer Progression
Source: Int J Mol Sci. 2023 Jan 11;24(2):1469. doi: 10.3390/ijms24021469 (PMC9865857; doi:10.3390/ijms24021469)
Supplement: Supplementary file 1 [file ijms-24-01469-s001.zip › ijms-2099754-supplementary.pdf]

J-I

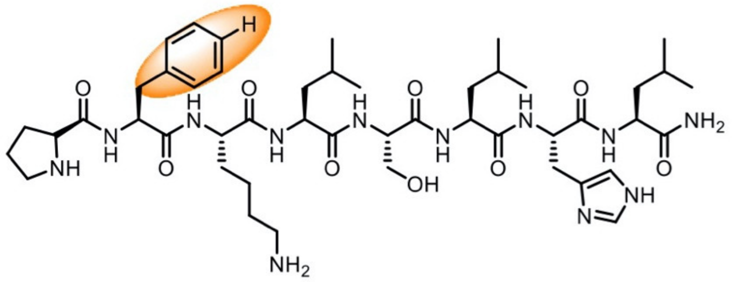

Cl-J-I

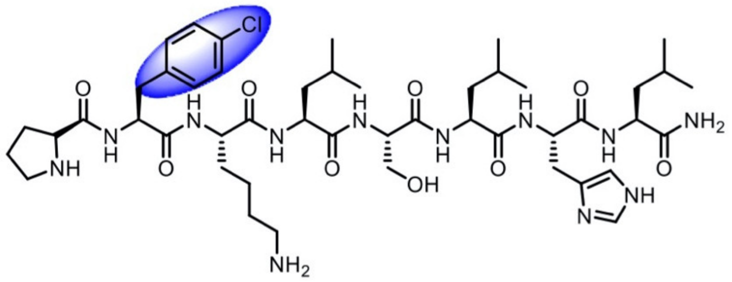

Br-J-I

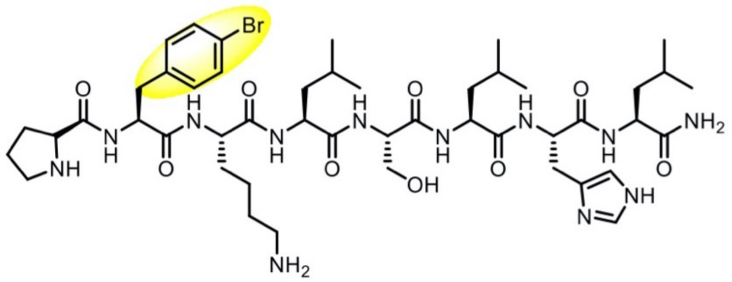

I-J-I

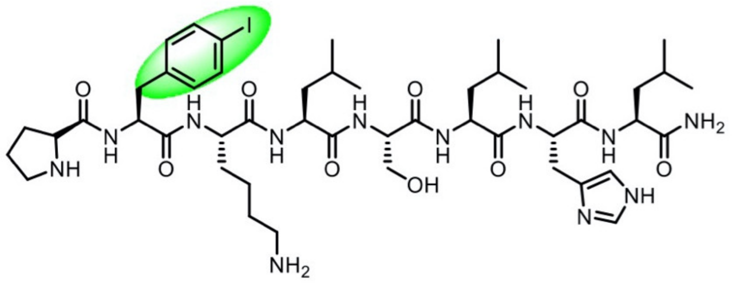

Supplemental Figure S1. **Structures of J-I and its derivatives Cl-J-I, Br-J-I and I-J-I shown the modifications of Phe with chlorine, bromine, and iodine, respectively.** These structures were drawn by ChemBioDraw software.

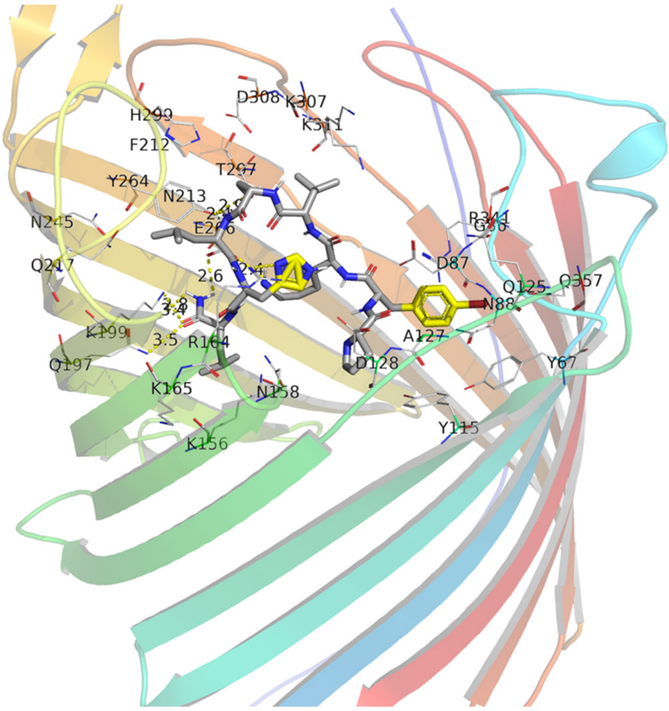

Supplemental Figure S2. Molecular modeling of Br-J-I binding to potential target FomA. Br-J-I interacts with extracellular portion of porin FomA (PDB extracted from AlphaFold database AF-Q47903) with the eight hydrogen bonds shown in yellow dashed lines (distance labeled in Å). FomA Br atom forms polar contact with N88 of long loop (L) 1 and Q357 of L8 with 3.9 Å and 3.2 Å, respectively. K3, S5, and H7 of Br-J-I form three hydrogen bonds with E266, Y264, and R164 of FomA, respectively. C-terminal carbonyl oxygen forms polar contacts with E197 and K199 of FomA. Nitrogen of C- terminal amide group forms a hydrogen bond with Y243 of FomA. For clarity, only the protein segments that contain the groups of interest are shown. FomA is shown in an α-carbon rainbow cartoon from blue in N-terminus to Red in C-terminus. The potential residues of FomA for interaction with Br-J-I are shown in lines and Br-J-I are in sticks. N, O, and C atoms of side chains are colored blue, red and gray, respectively. Br is in orange. Molecular docking was carried out by Vina docking software, and the interactions between ligand and receptor were analyzed and presented by PyMOL.

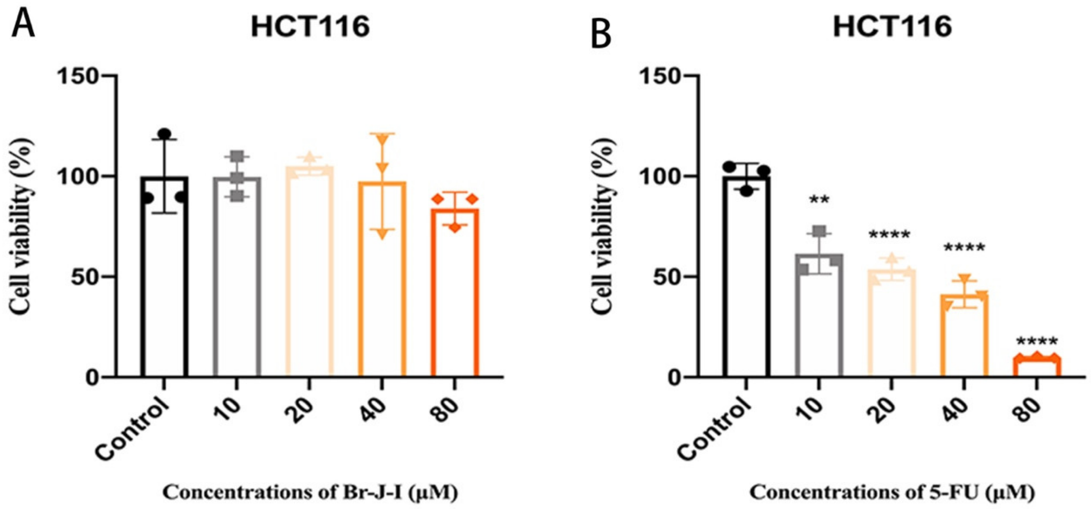

Supplemental Figure S3. Cell viability of CRC cells HCT116 in the presence of Br-J-I or 5-FU. Cell viability of human colon cancer cells HCT116 treated with different concentrations of Br-J-I (A) and 5-FU (B) for 72 h.

Supplemental Table S1. The primer sequences for qPCR.

Name	Primer sequences (forward primer)	Primer sequences (reverse primer)
16S	5′-GGTGAATACGTTCCCGG-3′	5′-TACGGCTACCTTGTTACGACTT-3′
Fn-specific-RNA	5′-CAACCATTACTTTAACTCTACCATGTTCA-3′	5′-GTTGACTTTACAGAAGGAGATTATGTAAAAATC-3′
FadA	5′-TGATGCAGCAAGTTTAGTAGGTGAA-3′	5′-TGCTAGTGCTTGTCTAGCAGCG-3′
Tnf	5′-AGGGTCTGGGCCATAGAACT-3′	5′-CCACCACGCTCTTCTGTCTAC-3′
Il1b	5′-GGTCAAAGGTTTGGAAGCAG-3′	5′-TGTGAAATGCCACCTTTTGA-3′
Claudin	5′-GTCTTTGACTCCTTGCTGAATCTG-3′	5′-CACCTCATCGTCTTCCAAGCAC-3′
Zo-1	5′-GGGAGGGTCAAATGAAGACA-3′	5′-GGCATTCCTGCTGGTTACAT-3′

Fn-specific-RNA as a target to quantitate *Fn* load of the tumor tissues of CRC mice with *Fn* colonization by quantitative PCR.
